# S1 guidelines “lumbar puncture and cerebrospinal fluid analysis” (abridged and translated version)

**DOI:** 10.1186/s42466-020-0051-z

**Published:** 2020-03-16

**Authors:** H. Tumani, H. F. Petereit, A. Gerritzen, C. C. Gross, A. Huss, S. Isenmann, S. Jesse, M. Khalil, P. Lewczuk, J. Lewerenz, F. Leypoldt, N. Melzer, S. G. Meuth, M. Otto, K. Ruprecht, E. Sindern, A. Spreer, M. Stangel, H. Strik, M. Uhr, J. Vogelgsang, K.-P. Wandinger, T. Weber, M. Wick, B. Wildemann, J. Wiltfang, D. Woitalla, I. Zerr, T. Zimmermann

**Affiliations:** 1 Fachklinik für Neurologie Dietenbronn, Dietenbronn 7, 88477 Schwendi, Germany; 2 Neurologische Uniklinik im RKU, Universitätsklinikum Ulm, Oberer Eselsberg 45, 89081 Ulm, Germany; 3 Praxis rechts vom Rhein, Böckingstr. 54-56, 51063 Köln, Germany; 4 Medizinisches Labor Bremen GmbH, Haferwende 12, 28357 Bremen, Germany; 5 Klinik für Neurologie mit Institut für Translationale Neurologie, Universitätsklinikum Münster, Albert Schweitzer Campus 1, 48149 Münster, Germany; 6 Klinik für Neurologie, Universitätsklinikum Ulm, Oberer Eselsberg 45, 89081 Ulm, Germany; 7 St. Josef Krankenhaus, Klinik für Neurologie und klinische Neurophysiologie, Asberger Str. 4, 47441 Moers, Germany; 8 Universitätsklinik für Neurologie, Medizinische Universität Graz, Auenbruggerplatz 22, A-8036 Graz, Austria; 9 Labor für Klinische Neurochemie, Uniklinik für Psychiatrie, Schwabachanlage 6, 91054 Erlangen, Germany; 10 Institut für Klinische Chemie, Klinik für Neurologie, Universitätsklinikum Schleswig-Holstein Kiel, Arnold-Heller-Str. 3, 24105 Kiel, Germany; 11 Klinik für Neurologie, Charité - Universitätsmedizin Berlin, Charitéplatz 1, 10117 Berlin, Germany; 12 DIAKOVERE Friederikenstift, Humboldt-Str. 5, 30169 Hannover, Germany; 13 Klinik für Neurologie, Universitätsklinikum Mainz, Langenbeckstr. 1, 55131 Mainz, Germany; 14 Klinische Neuroimmunologie und Neurochemie, Klinik für Neurologie, Medizinische Hochschule Hannover, Carl-Neuberg-Str. 1, 30625 Hannover, Germany; 15 Klinik für Neurologie, Klinikum der Sozialstiftung Bamberg, Buger Straße 80, 96049 Bamberg, Germany; 16 MPI für Psychiatrie München, Kraepelinstr. 2, 10 80804 Munich, Germany; 17 Klinik für Psychiatrie und Psychotherapie, Universitätsmedizin Göttingen, Von-Siebold-Str. 5, 37075 Göttingen, Germany; 18 Institut für Klinische Chemie und Klinik für Neurologie, UKSH Campus Lübeck, Ratzeburger Allee 160, 23528 Lübeck, Germany; 19 Klinikum Stephansplatz, Stephansplatz 3, 20354 Hamburg, Germany; 20 Institut für Laboratoriumsmedizin, Klinikum der LMU, Marchioninistr. 15, 81377 Munich, Germany; 21 Klinik für Neurologie, Universität Heidelberg, Im Neuenheimer Feld 400, 69120 Heidelberg, Germany; 22 Neurologische Klinik, Katholische Kliniken der Ruhrhalbinsel, Heidbergweg 22-24, 45257 Essen, Germany; 23 Neurologische Klinik, Universitätsmedizin Göttingen, Robert-Koch-Str. 40, 37075 Göttingen, Germany; 24 Labopart - Medizinische Laboratorien, Wurzener Str. 5, 01127 Dresden, Germany

## Abstract

**Introduction:**

Cerebrospinal fluid (CSF) analysis is important for detecting inflammation of the nervous system and the meninges, bleeding in the area of the subarachnoid space that may not be visualized by imaging, and the spread of malignant diseases to the CSF space. In the diagnosis and differential diagnosis of neurodegenerative diseases, the importance of CSF analysis is increasing. Measuring the opening pressure of CSF in idiopathic intracranial hypertension and at spinal tap in normal pressure hydrocephalus constitute diagnostic examination procedures with therapeutic benefits.

Recommendations (most important 3-5 recommendations on a glimpse):

1. The indications and contraindications must be checked before lumbar puncture (LP) is performed, and sampling CSF requires the consent of the patient.
2. Puncture with an atraumatic needle is associated with a lower incidence of postpuncture discomfort. The frequency of postpuncture syndrome correlates inversely with age and body mass index, and it is more common in women and patients with a history of headache. The sharp needle is preferably used in older or obese patients, also in punctures expected to be difficult.
3. In order to avoid repeating LP, a sufficient quantity of CSF (at least 10 ml) should be collected. The CSF sample and the serum sample taken at the same time should be sent to a specialized laboratory immediately so that the emergency and basic CSF analysis program can be carried out within 2 h.
4. The indication for LP in anticoagulant therapy should always be decided on an individual basis. The risk of interrupting anticoagulant therapy must be weighed against the increased bleeding risk of LP with anticoagulant therapy.
5. As a quality assurance measure in CSF analysis, it is recommended that all cytological, clinical-chemical, and microbiological findings are combined in an integrated summary report and evaluated by an expert in CSF analysis.

**Conclusions:**

In view of the importance and developments in CSF analysis, the S1 guideline “Lumbar puncture and cerebrospinal fluid analysis” was recently prepared by the German Society for CSF analysis and clinical neurochemistry (DGLN) and published in German in accordance with the guidelines of the AWMF (https://www.awmf.org). /uploads/tx_szleitlinien/030-141l_S1_Lumbalpunktion_und_Liquordiagnostik_2019-08.pdf). The present article is an abridged translation of the above cited guideline. The guideline has been jointly edited by the DGLN and DGN.

## Introduction

The present article is an abridged translation of the guideline recently published online (https://www.awmf.org/uploads/tx_szleitlinien/030-141l_S1_Lumbalpunktion_und_Liquordiagnostik_2019-08.pdf). This guideline contains basic recommendations concerning practical procedures for CSF space puncture, in particular with regard to indications and possible contraindications, information and consent, selection of the puncture needle, procedure in patients treated with anticoagulants, and thrombocytic function inhibitors, sample collection, treatment, and analysis as well as for compiling findings. The long version deals in detail with individual clinical presentations which had to be shortened for reasons of space in the present guideline. The structure, table of contents, and basic features are presented here.

## Diagnostic lumbar puncture

### Indications

Apart from a brain biopsy, CSF analysis is the only procedure that can detect inflammation in the CSF or the central nervous system. Therefore, meningitis, encephalitis, myelitis, radiculitis, and (poly)neuritis in acute or chronic form constitute core indications for lumbar puncture (LP) (Table 1). CSF analysis is playing an increasingly important role in neurodegenerative diseases, especially for dementia and differential diagnoses. Detecting malignant cells in the CSF confirms the diagnosis of a meningeosis carcinomatosa or lymphomatosa. The detection of blood and its degradation products in the CSF can confirm the diagnosis of subarachnoid hemorrhage even if the diagnosis cannot be made by cranial CT. LPs for relief in normal pressure hydrocephalus or idiopathic intracranial hypertension represent a special case. In children under 18 years of age, fever of unknown cause was the most frequent indication for LP at 20%, in adult patients headache at 39% [45, 179].

**Table 1:** Indications for the diagnostic LP under consideration of the contraindications (see below)

Suspected condition	
…Meningitis	
…Encephalitis	
…Myelitis	
…Neuroborreliosis	
…Neurotuberculosis	
...Polyradiculoneuritis Guillain-Barré	
…Chronic inflammatory demyelinating polyneuropathy	
…Encephalomyelitis disseminata	
…Neuromyelitis optica spectrum disorder	
…Neurosarcoidosis	
…Neurolupus	
…Subarachnoidal hemorrhage	
…Meningiosis carcinomatosa	
…Meningiosis lymphomatosa	
...Idiopathic intracranial hypertension	
...Normal pressure hydrocephalus	
Differential diagnosis of the following core symptoms:	
-Headache	
-Dementia syndrome	
-Sepsis with unknown focus of infection	

### Contraindications

#### Increased intracranial pressure

Before electively collecting CSF, the presence of clinical CSF pressure signs must be ruled out. Cranial imaging (CCT, cMRT) prior to LP is needed in special cases (clinical evidence of increased cerebral pressure, focal neurological deficits, first epileptic seizure, vigilance disorder, or history of immunosuppression), but is not necessary in the absence of clinical signs of increased cerebral pressure. The removal of CSF in cases of increased cerebrospinal pressure can lead to an entrapment of neuronal structures due to axial displacement of the brain and may be fatal. Examination of the ocular fundus by ophtalmoscopy is less sensitive than cross-sectional imaging as a congestive papilla may be absent despite increased intracranial pressure. The presence of a congestive papilla in the case of idiopathic intracranial hypertension does not represent a contraindication for a relief puncture.

#### Tendency to bleed

A platelet count below 50,000/μL, a quick test below 50%, an INR of more than 1.8, and a clearly pathologically activated partial thromboplastin time (aPTT) are considered contraindications for LP. When in doubt, the platelet aggregation time can be determined by apparatus or the bleeding time can be clinically determined by a scratch test.

Thrombopenia below 50,000/μL is a relative contraindication and thrombopenia below 10,000/μL an absolute contraindication. In the case of thrombocyte counts below 10,000/μL, thrombocytes should always be substituted prior to LP. In the range between 10,000 and 50,000/μL an increased complication rate is to be expected. The decision for thrombocyte substitution must be made individually.

Therapeutically induced coagulation disorders should - if medically justifiable - be stopped before the procedure, and their effect should be eliminated by medication if necessary (Table 2).

**Table 2:** Recommendations for diagnostic LP in thrombocytopenia (see also cross-sectional guideline of the Bundesärztekammer on therapy with blood components and plasma derivatives (https://www.bundesaerztekammer.de/fileadmin/user_upload/downloads/pdf-Ordner/WB/QLL_Haemotherapie-englisch.pdf))

Thrombocyte count /μL	Procedure for a planned LP
> 50,000	If there are no other contraindications.
10,000–50,000	Relative contraindication. An increased risk of bleeding is to be expected. Substitute thrombocytes if necessary.
< 10,000	Absolute contraindication. Thrombocyte substitution before LP mandatory.

Patients anticoagulated with Phenprocoumon or other coumarin derivates should be transitionally switched to heparin, as this can be antagonized more rapidly. At this point we would like to refer to the S1 guideline of the DEGAM (German Society for General Medicine and Family Medicine) on the subject of bridging (AWMF 053/027 [111, 122];). In emergencies, an attempt to normalize the blood coagulation can be undertaken by substituting coagulation factors. This also applies to individuals with a disease-related lack of clotting factors.

For the use of NOAK (new oral [or non-vitamin K-dependent] anticoagulants) such as dabigatran, rivaroxaban, apixaban, and edoxaban for the prophylaxis or therapy of thromboembolic events no systematic studies are available. Initial recommendations [39] consider emergency punctures under therapy in vital indications. Elective punctures of the CSF space should be carried out - if medically justifiable - after interrupting the NOAK according to the respective half-life, considering renal function, in particular for dabigatran (usually 2-3 days, for dabigatran and GFR [glomerular filtration rate] below 50 ml/min > 3 days). For the treatment of life-threatening bleeding, idarucizumab is available in Germany as a specific antidote that antagonizes the effects of the thrombin inhibitor dabigatran by picking up the Fab antibody fragment (Fab: “fragment antigen binding”).

Andexanet alfa has already been approved and is available as an antidote for bleeding caused by Factor Xa inhibitors. Approval was granted in the first half of 2019 in Germany. If discontinuation of NOAK is associated with increased thromboembolic risk, conversion to heparin (bridging) is recommended [39, 111].

A case of a bleeding complication after LP under a double platelet inhibition with ASS (acetylsalicylic acid) and clopidogrel has been reported [137]. Systematic studies on the frequency of bleeding complications after LP in patients with dual thrombocyte aggregation inhibition (dTAH) are missing, however. For individuals with dTAH and planned LP in emergency indications and high thrombotic risk, the LP should be carried out while maintaining the dTAH according to a recommended procedure [39]. In the case of elective LP and high thrombotic risk a delay of the LP should be considered. For low thrombotic risk, elective LP is postponed 1 week after discontinuing clopidogrel under aspirin monotherapy.

ASS does not need to be discontinued for LP.

#### Infection in the course of the puncture pathway

Both superficial and deep inflammation of the skin or subcutis, but also inflammation of the muscle in the area of the puncture site represent a contraindication for LP.

#### Lack of consent in a patient who is able to give consent

Here, the risk of the intervention must be weighed against the potential benefit.

#### Lack of consent for emergency indications

In emergency situations (for example, acute bacterial meningitis is clinically suspected), which cannot be delayed, the LP can also be carried out without a declaration of consent from patients who are unable to consent. It is recommended to document this consideration in written form.

#### Pregnancy

The benefit of the diagnostic measure must be weighed against the additional risk of inducing premature labor. In cases of idiopathic intracranial hypertension (IIH), relief punctures with reduction in visual acuity are among the therapeutic options available, even during pregnancy [78, 166].

### Implementation

#### Informing the patient

Outside the scope of individual case decisions (e.g., emergency indication for patients who are not capable of giving consent), a patient who is capable of giving consent or the legal representative of a patient who is not capable of giving consent is required for LP. The information should always be provided in written form and patients should be given sufficient time for reflection. The procedure differs depending on the indication for the puncture and is also dependent on the patient’s level of consciousness. If an appropriate reflection period cannot be adhered to for clinical reasons, this must be noted separately. In this case, the physician performing the procedure must also document the indication. The patient can waive a further reflection period in written form.

The information for the patient should include the following:

- Information about risk and benefit.
- Adverse consequences if LP is not carried out, depending on the respective suspected diagnosis.
- Identification of alternative diagnostic methods.
- Explanation of the technical aspects of the puncture:
  - Procedure of the examination.
  - Possibility of local anesthesia. If a local anesthetic is used, possible hypersensitivity reactions must always be pointed out.
- Indications of possible adverse effects.

It should also be pointed out that patients may need to be hospitalized and the inpatient stay extended if side effects develop. In exceptional cases it may be necessary to perform a second puncture (with blood patch); in very rare cases, surgical measures may be necessary to treat complications (e.g., subdural hematoma).

If a suboccipital puncture is to be performed, additional reference to this should be made:

- Possible occurrence of a centrally caused circulatory or respiratory disorder
- Possible occurrence of suboccipital hemorrhage with atypical course of an arterial vessel (owing to this complication the suboccipital puncture approach is no longer routinely carried out)
- Information on suboccipital puncture should include the alternative of other puncture routes

For purposes of clarification, ready-made information sheets are commercially available.

The LP can be performed in an outpatient or inpatient setting after the patient has been informed in detail about benefits, procedures, and risks and after the patient has provided documented consent. In addition to the severity of the clinical presentation, patient-related factors such as age, weight, comorbidities, and coagulation status; organizational aspects such as the availability of the examination procedure; further CSF analysis; and the patient’s wishes also play a role in deciding whether the LP should be performed in an outpatient or inpatient setting. In advance, it must be checked whether special precautions must be taken to ensure that the CSF is properly processed, e.g., information about the laboratory or the laboratory courier in order to guarantee a prompt cell count or cytological processing. Preanalytics also play a role, for example, when CSF samples need to be freshly stained for microscopy. The container in which the CSF is collected and stored also affects the results: Proteins that tend to form aggregates, for example, amyloid-β1-42, are particularly highly absorbed by certain tube materials such as glass or polystyrene, resulting in false-positive results in Alzheimer’s disease diagnostics [42]. The use of polypropylene tubes is therefore recommended. It should also be noted that the sample containers selected should be made of the same material, at least within one center, from LP to laboratory analysis (including for aliquoting, biobanking, etc.).

As a rule, CSF is drawn by LP. The selection of the puncture needle depends on the anatomical conditions and the experience of the examiner. If possible, an atraumatic puncture needle should be used in order to minimize postpuncture syndrome [128]. A sharp needle is preferred in older or obese patients, also for expected difficult punctures, if necessary, and as a rule for measuring pressure and for attempted drainage.

### Technical implementation of the LP

#### General information

The puncture should be performed by or under the supervision of an experienced physician. Standards for disinfection and hygiene must be met [147]. These include:

- The physician must wear sterile gloves.
- A sterile drape or cover must be used.
- The skin must be locally disinfected using a sterile swab and include at least one preliminary cleansing step. The exposure time of the disinfectant as specified by the manufacturer must be considered.
- Suitable measures should be taken to prevent contamination of the cannula. These include:
  - Handling under sterile conditions
  - Avoidance of contact with the patient’s clothing or exam table cover.

In the literature, the need for wearing a face mask while performing an LP has been the subject of controversy [10, 56, 121, 155]. Prospective studies to address this question have not been carried out, but numerous case reports of iatrogenically induced meningitis have been published. Molecular genetics investigations have proven that the infection was caused by microbes found in the oral cavity of the physicians [177]. These case reports indicate that the incidence of iatrogenic infections increases with the injection of diagnostic (myelography) or therapeutics (chemotherapy, local anesthesia). In such cases the KRINKO (Commission for hospital hygiene and infection prevention) recommends more intensive preventive measures such as the use of mouth-nose masks by the physician and the assisting personnel.

From a pathogenetic point of view, an iatrogenic infection appears more likely if the physician has a respiratory infection and if talking while performing an LP [10].

Overall, the risk of iatrogenic infection in diagnostic punctures is low. Nevertheless, a face mask should be worn under the following conditions:

- Presence of a respiratory infection in the physician, the assisting staff, or the patient
- Injections into the CSF space
- LP under training conditions (accompanied by explanations or instructions).
- Implementation of further diagnostic measures (e.g., CSF pressure measurement) with increased time expenditure.
- Suspicion of an aerogenic infection (e.g., meningococcal meningitis) of the patient for self-protection.
- In all other cases, consideration should be given to whether the physician should also wear a face mask as little effort is involved and the potential benefit is substantial.

#### Local anesthesia

Decisions concerning local anesthesia must be made individually, but it is not generally recommended. If necessary, about 2 ml of a 1-2% lidocaine solution should be given for local anesthesia and it should be administered close to the skin surface. Puncture of the spinal canal must be avoided.

#### CSF pressure measurement

If an indication to measure CSF opening pressure is given, it should be measured, before a CSF sample is drawn. CSF pressure must be measured with the patient in the horizontal recumbent position. If this is not initially possible, LP can first be performed with the patient in a sitting position, but the CSF pressure must still be measured with the patient lying down. It is also important to ensure that the environment is sterile.

The reference values for CSF pressure with the patient lying down are as follows:

100-250 mmH_2_O (2.5 and 97.5 percentiles [188];. The CSF pressure is dependent on the BMI [188]. Pulsations of 2-5 mm, in lying position of 4-10 mm, occur synchronously with the pulse.

To assure quality standards in CSF analysis, a standardized amount of CSF should be collected (10-15 ml) as a concentration gradient of CSF protein develops (the protein concentration is higher in the first fraction of CSF taken than in the last fraction [145, 167]. The quantity of the extracted CSF has no influence on whether postpunctural headache symptoms develop [100]. In individual cases (tuberculosis diagnosis, FACS analysis [FACS: fluorescence-activated cell sorting]) up to 30 ml CSF can be drawn without increasing the risk of complications [124].

The CSF sample should subsequently be collected in 3 different tubes if the first CSF sample contains blood in order to distinguish artificial contamination from pathological bleeding [124].

#### Puncture site

The LP is performed between the 3rd and 5th lumbar vertebral body (LWB). A puncture above LWB 2/3 should be avoided due to anatomical conditions (the conus medullaris extends to LWB 1/2 in 94% of the cases).

Spinal tap can be performed with the patient either lying or sitting. For CSF pressure measurement see the section above. During LP a kyphosis of the lower spine is desirable. It is preferable to perform LP with the patient in a sitting position if CSF pressure is not measured and the patient is awake and cooperative. This is more comfortable (faster and more accurate) as the anatomical situation of the spine is clearer.

Suboccipital puncture should only be performed in exceptional cases, when in emergency situations no CSF can be obtained by LP, or pathologically anatomical conditions (e.g., local abscess) represent a contraindication for LP.

#### Risks, side effects, and complications

Frequent side effects (> 3%) include:

- Local pain at the puncture site
- Acute transient lumbar radicular irritation symptoms
- Light bleeding locally
- Postpuncture syndrome

A postpuncture syndrome is an orthostatic headache, which can occur after LP especially if performed in an upright position. It may be accompanied by nausea, vomiting, and sensitivity to light [37]. For treatment of postpuncture headache, we refer to the AWMF S1 guideline 030/113 “Diagnostics and Therapy of the postpuncture and spontaneous CSF negative pressure syndrome” [36].

In a case series at a hospital in rural Congo, 307 consecutive patients with LP were treated with a complication rate of 7.5%, namely, headaches, back pain, and confusion. All side effects were transient in nature, and no permanent damage was observed [125].

Rare complications (< 3%) include:

- Infection of the injection canal
- Circulatory reactions, which can be as serious as syncope

In individual cases the following complications have been reported:

- Bleeding with neurological deficits, mainly when LP was performed despite contraindications or if vascular anomalies were present
- Subdural hematomas
- Cranial nerve palsies
- Migraine attacks
- Epileptic seizures
- Entrapment syndromes, mainly when contraindications were disregarded

#### Reporting

All findings of the CSF analysis, including inspection, cell count, cytology, immunocytochemistry if necessary, and ranging from protein analytics to microbiological findings, should be presented in an integrated report that is summarized and checked for plausibility. The following chapters give general (Section Basic cerebrospinal fluid diagnostic testing) and -specific (Sections Infectious inflammatory diseases, Non-Infectious inflammatory diseases, Degenerative Disorders, Vascular diseases, Neoplastic diseases, Other) guidance on how summary reports should be prepared.

## Basic cerebrospinal fluid diagnostic testing

**Fig. 1:**
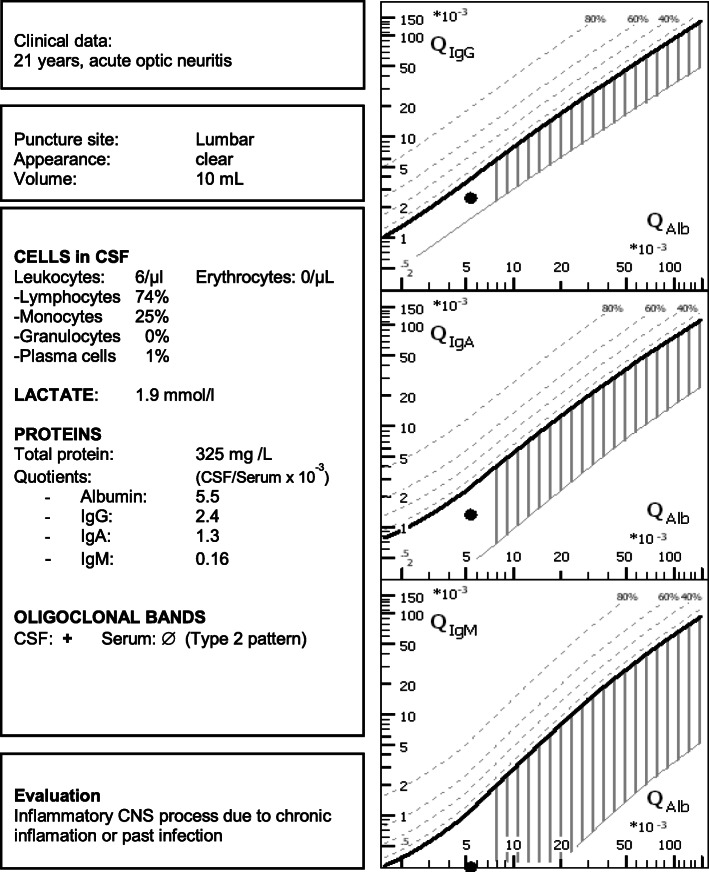
Example of an integrated total CSF report

- CSF analysis requires an assessment of all individual findings that should be summarized in an integrated, overall report so as to present findings that are reliable and diagnostically meaningful.
- It is important to specify a meaningful question.
- By applying an integrated diagnostic strategy, on the one hand, typical disease patterns can be identified and, on the other, plausibility checks can help avoid analytical errors (Fig. 1).

CSF examination consists of a three-step process (Table 3).

**Table 3:** Steps of CSF analysis

Analytical step	Parameters	Indication/Remarks
Emergency analysis	Appearance (if contaminated with blood, 3-tubes test), cell count, total protein, lactate	Acute inflammation, bacterial or viral, cerebral bleeding (SAH, ICH)
Basic analysis	Quotients Albumin, IgG, IgA, IgM, and oligoclonal bands	Intrathecal inflammation, Blood-CSF barrier function
	Differential cell count	Differentiation of inflammation, bleeding, and neoplastic involvement
	Gram staining and culture	Pathogen detection (bacteria, fungi)
Extended analysis	Pathogen-specific antibody (Antibody index)	Infection or autoimmune disease
	CNS-specific proteins	Neurodegenerative diseases (AD, CJD, ALS, Narcolepsy, etc.)
	Immune cytology, tumor markers	Tumor: confirmation and subtyping
	Antigen detection	Pathogen detection (bacteria, fungi)
	PCR	Standard for viruses and Tbc, some other bacteria and parasites^a^

*AD* Alzheimer’s disease, *ALS* amyotrophic lateral sclerosis, *CJD* Creutzfeldt-Jakob disease, *ICH* intracerebral hemorrhage, *SAH* subarachnoid hemorrhage, *Tbc* tuberculosis

^a^ e.g., if findings from staining and antigen detection are negative

The reference ranges for routine parameters [140, 173] are summarized in Table 4.

**Table 4:** Reference ranges for routine parameters

Parameter	Method	Reference range
Appearance	Inspection	clear, colorless
Cell count (Leukocytes/μL)	Manual evaluation by light microscopy, Fuchs-Rosenthal chamber	< 5
Differential cytology staining	Manual evaluation by light microscopy, Pappenheim staining	Lymphomonocytic (ratio 2:1 bis 3:1)
Total protein (mg/L)	Nephelometry/Turbidimetry	< 500
L-Lactate (mmol/L)	Enzymatic	0.9 – 2.7 (age dependent) [102]
Glucose (L/S)	Enzymatic	> 0.50
Albumin (L/Sx10^−3^)	Nephelometry	< 5 - 10 (age dependent)
Ig-Synthesis in CNS	Nephelometry	Not detectable
Pathogens	Gram staining, culture, light microscopy, PCR, antigen detection	Not detectable
Pathogen-specific antibody (intrathecal synthesis)	Enzyme immunoassays	Not detectable
Brain-specific proteins (pg/mL)	Enzyme immunoassays	Tau-Protein (< 450) Phospho-Tau (< 60) Abeta1-42 (> 550) (Laboratory- and assay dependent ranges) Abeta1-42/Abeta1-40-Quotient (> 0,1)

L/S = CSF/Serum quotient

### Cytology

Normal CSF contains less than 5/μL of nucleated cells composed of lymphocytes and monocytes in a ratio of 2:1 to 3:1 [191]. If CSF contains blood (artificial or subarachnoid hemorrhage), the erythrocytes are counted and reported separately [172]. Differential cytology by microscopy should be performed thoroughly at each puncture regardless of the total cell count. Automated cell counting and cell differentiation machines should be avoided in CSF analysis as the findings are not reliable [191].

#### Quantitative evaluation of intrathecally produced immunoglobulins

In order to be able to establish whether immunoglobulins or pathogen-specific antibodies are being produced intrathecally, CSF and blood need to be examined in parallel as the largest protein fractions in the CSF originate from the blood. These CSF-blood quotients are related to the individual blood-CSF barrier function (albumin-CSF/serum quotient, QAlb) [140, 173].

**Fig. 2:**
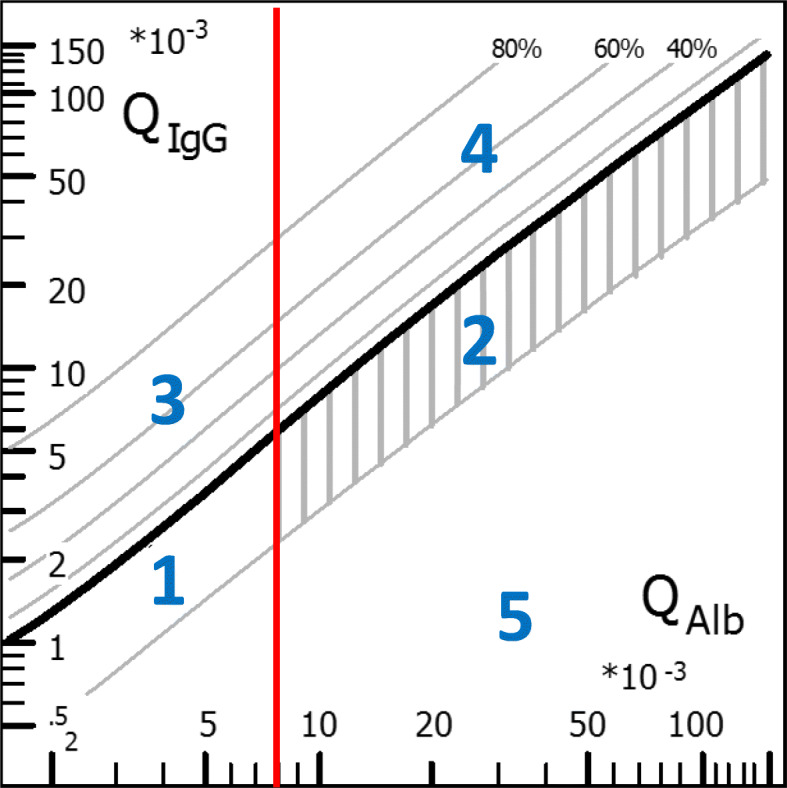
Quotient diagram. Logarithmically the albumin quotient is plotted against the IgG quotient. The thick diagonal line represents the QLim. This corresponds to the mean value of the expected IgG concentration plus 3 times the standard deviation. For IgG quotients above this line, an intrathecal IgG synthesis can therefore be assumed with a probability of a false-positive result of < 0.5%. The red vertical line represents the age-related limit value for barrier function (formula: age/15 + 4, the upper normal range of a 60-year-old person would be 8 × 10^− 3^). This results in different areas with differently interpreted findings (and disease examples). An advantage of the quotient diagrams over a numerical calculation is that typical finding constellations can be assigned to a disease at a glance: Possible constellations resulting from QIgG and QAlb are: (1) Normal findings, e.g., no indication of inflammatory CNS process. (2) Isolated barrier dysfunction, e.g., Guillain-Barré syndrome or spinal canal stenosis. (3) Isolated inflammation in the CNS, e.g., multiple sclerosis or past infectious encephalitis. (4) The combination of (2) and (3), e.g., acute neuroborreliosis, neurotuberculosis. (5) Implausible findings (e.g., high-dose hook effect, puncture soon after immunoglobulin infusion)

Albumin serves as a reference protein for the blood-CSF barrier as it originates exclusively from the blood. A corresponding graphic representation of the quotients was established by Reiber and Felgenhauer (Fig. 2) [145, 175].

Further advantages are that the quotient diagram can also be extended to IgA and IgM diagrams, so that three immunoglobulin classes can be evaluated in parallel, which increases the diagnostic significance of these parameters (see also Fig. 1 [175]).

#### Oligoclonal IgG bands

**Fig. 3:**
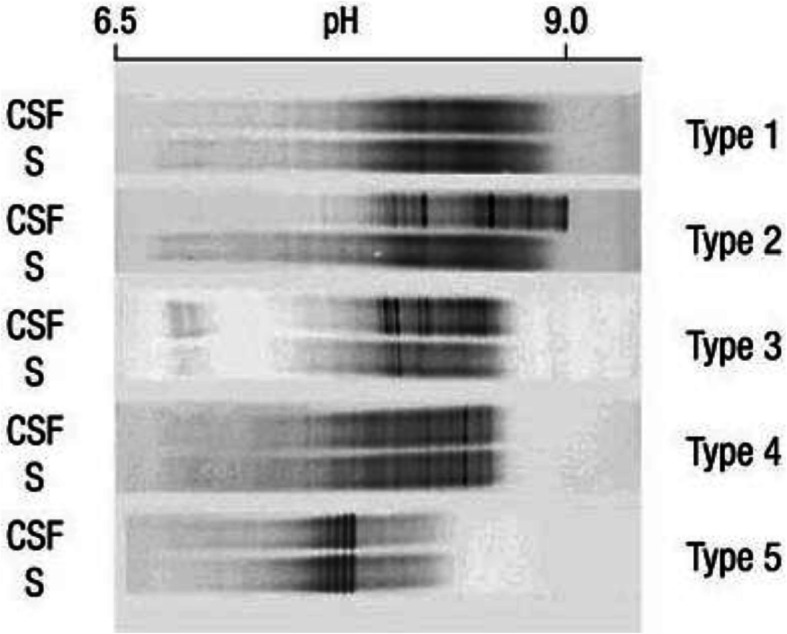
IgG band patterns. Five different patterns can be found, with patterns 2 and 3 indicating intrathecal synthesis, as shown in Fig. 3: Type 1: Normal finding. Type 2: Isolated OCB in the CSF. Type 3: Identical OCB in cerebrospinal fluid and serum, additionally isolated OCB in the CSF. Type 4: OCB with identical (mirror image) distribution in the CSF and serum. Type 5: Monoclonal bands (usually identical distribution in the CSF and serum) as an indication of systemic gammopathy [51]

**Fig. 4:**
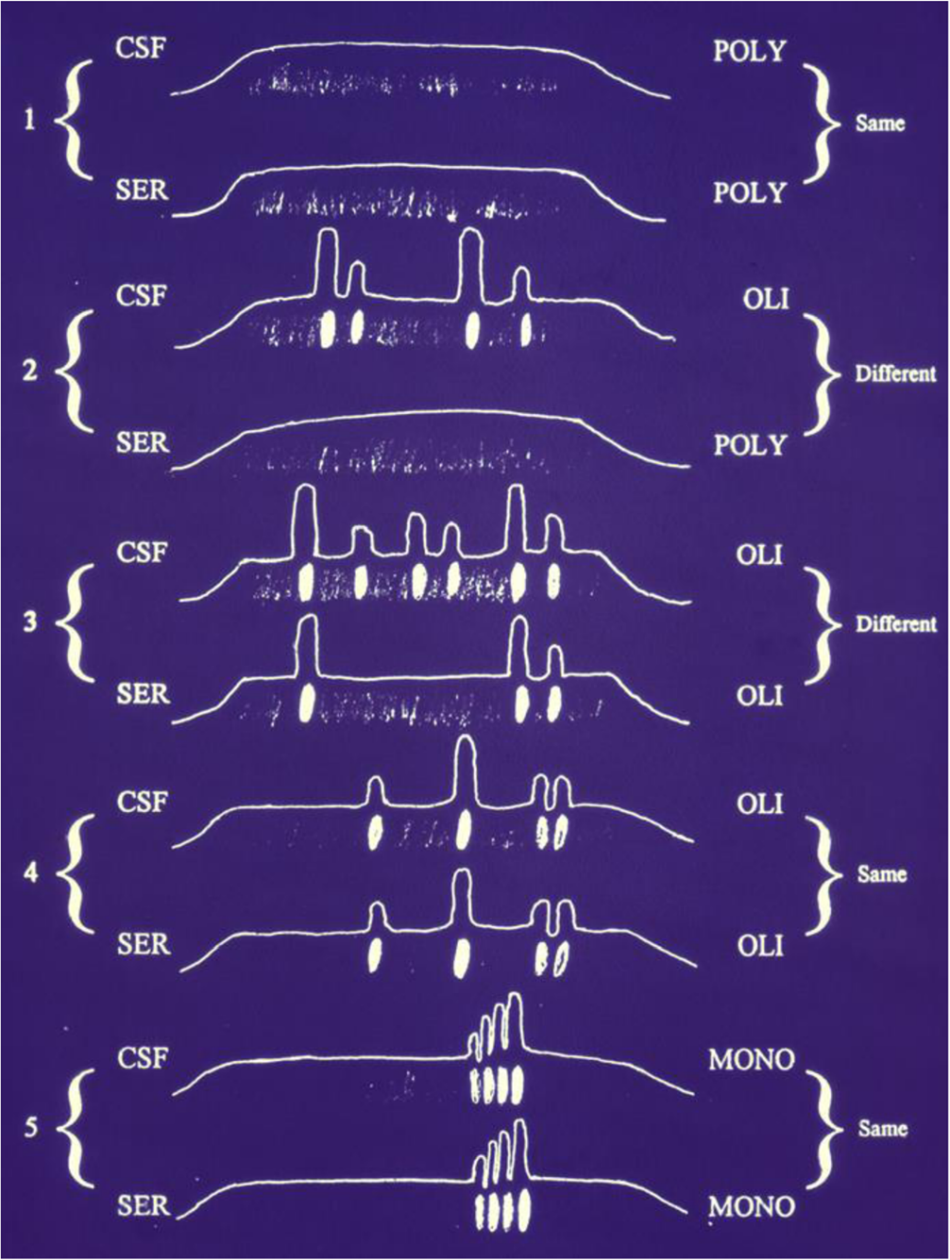
IgG band patterns illustrated graphically. Abbrev.: CSF, cerebrospinal fluid;, Ser, serum; Poly, polyclonal; Oli, oligoclonal; Mono, monoclonal [7]

Oligoclonal IgG bands (OCB) occur nonspecifically in subacute and chronic inflammatory diseases of the CNS. OCB are more sensitive than quantitative quotient diagrams to detect intrathecal IgG production. An OCB pattern is present when at least two CSF-specific bands are detected (Figs. 3 and 4) [140, 173].

## Infectious inflammatory diseases

### Bacterial meningitis

If bacterial meningitis is suspected clinically, CSF analysis is necessary. With regard to the clinical presentation and therapy, reference is made to the AWMF guideline 030/089 “Outpatient acquired bacterial (purulent) meningoencephalitis in adulthood” [142] and the “ESCMID guideline: diagnosis and treatment of acute bacterial meningitis” [17]. If cerebral imaging is necessary before LP is performed or if the LP is delayed, e.g., by cerebral imaging, empirical antibiotic therapy should be started beforehand. Antibiotic treatment reduces the sensitivity of methods for detecting bacterial pathogens. For this reason, blood cultures should always be obtained before antibiotics are administered [17, 67].

The “typical” CSF constellation of bacterial meningitis with granulocytic pleocytosis > 1000 cells/μL, total protein > 1000 mg/l and lactate > 3.5 mmol/L L is present in approx. 80% of cases. Depending on the pathogen, however, “atypical” findings can be seen in up to 25% of cases (see Table 5). If bacterial meningitis is not yet treated with antibiotics, an increased lactate value in the CSF is more sensitive than the cell count. Antibiotic therapy should still be administered when cell counts are low, but lactate or protein values are high in the CSF, however. Here, the possibility of “apurulent meningitis” must be considered.

**Table 5:** Overview of diagnostically relevant routine parameters in acute bacterial meningitis (BM) and frequency of “typical” and “atypical” changes depending on the bacterial pathogen

Parameter	Diagnostic (first) LP	Remarks / Special features
Cell count (CC) / μLμL	„typical "CC ≥1000: ca. 80% Mean (SD): 7753 (14736) CC 100–999: 14% CC < 100: 7%	[16] Cave „apurulent BM": do not discontinue antibiotic treatment when CC is low, but CSF lactate or protein values are high!
*S. pneumoniae*	Median (IQR): 1842 (291–4419) CC > 999: 75.8-78% CC < 100: 17-19.3% CC < 10: 5%	([17, 136]) (*n* = 153) [24];
*N. meningitidis*	Median (IQR): 5328 (1590–12.433) CC > 999: 80-82% CC 100–999: 6.5-11%; CC < 100: 9-11.6%, initial CSF normal: 1.7% (CC ≤5/μL, TP ≤0.50 g/l und Glucose ratio CSF / blood ≥0.40)	[68] (*n* = 258) [24];
*L. monocytogenes*	Median (IQR): 680 (291–1545) CC < 100: 11%	[99] (*n* = 30 + 62)
*H. influenzae* b	Median (Min-Max): 1470 (0–11,400) CC > 999: 92.9%; CC 100-999: 7% CC < 100: 0%	[14] (*n* = 11) [24]
B-streptococci	Median (Min-Max): 1230 (0-80,000) CC „normal": 6%	[57] (*n* = 242)
Newborn meningitis	CC ≤ 3: 10%	[54]
Differential Cell count	„typical "= granulocytic	
*S. pneumoniae*	≤20% Granulocytes: 5.9%	[24]
*N. meningitidis*	≤20% Granulocytes: 8.2%	[24]
*L. monocytogenes*	< 50% Granulocytes: 26%	[99]
*H. influenzae* b	≤20% Granulocytes: 4.3%	[24]
B-Streptococcus	Median (Min-Max): 87 (0-100) %	[57]
Total protein (TP) in mg/l	„typical "TP > 1000 Mean (SD): 4900 (4500)	[16]
*S. pneumoniae*	Median (IQR): 2700 mg/l (1400–5800)	[136]
*N. meningitidis*	Median (IQR): 4500 mg/l (2200–7000)	[68]
*L. monocytogenes*	Median (IQR): 2500 mg/l (1760–3650)	[99]
*H. influenzae* b	Median: 1800 mg/l	[14](*n* = 11)
B streptococci	Median (Min-Max): 2480 mg/l (200-16,000)	[57]
Newborn meningitis	TP < 400: 0% TP 410-1200: 24% TP > 1200: 76%,	[54]
Lactate (mmol/L)	„typical "Lactate ≥3.5 l diverging reference ranges, recommended cut-off: 3.9 mmol/L (=35 mg/dl) Man (SD): 16.51 (6.1) Median (IQR): 9.9 (6.8-12.9) mmol/L	[1, 97, 152]
DD bacterial versus viral meningitis	Lactate as sensitive differentiation criterion (Meta-analysis: [79, 152]) Sensitivity untreated BM: 98% Sensitivity after preantibiosis: 49%	Cave: Lactate increase also found in status epilepticus, cerebral infarction, ICB, Tumor, Herpes encephalitis [152]
Macroscopy / Gram staining	positive 63-72% no preantibiosis: 63% with preantibiosis: 62%	[24] (*n* = 667); [130]
*S. pneumoniae*	positive: 85.2%	[24] (*n* = 162)
*N. meningitidis*	positive: 72.5-89%	[24] (*n* = 356) [68]; (*n* = 244)
*L. monocytogenes*	positive: 37%	[99]
*H. influenzae* b	positive: 83.3%	[24] (*n* = 72)
CSF culture	Positive no preantibiosis: 65.8-88%, positive with preantibiosis: 61.4-70%	[24, 130]
*S. pneumoniae*	positive: 75-87%	[96] (*n* = 83) [24];
*N. meningitidis*	positive: 79.5%	[24]
*H. influenzae* b	positive: 50%	[24]
Blood culture	positive no preantibiosis: 66% positive with preantibiosis: 48%	[16, 130]
*S. pneumoniae*	positive: 42.6-67%	[24, 96] (*n* = 76) [136]; (*n* = 186)
*N. meningitidis*	positive: 12.6 -57%	[24, 68] (*n* = 227)
*L. monocytogenes*	positive: 61%	[99]
*H. influenzae* b	positive: 50%	[24]
Newborn meningitis	positive: 62%	[54] (*n* = 92)

Owing to currently available multiplex systems for nucleic acid amplification, the most frequently observed meningitis and encephalitis pathogens can be rapidly investigated in a CSF sample. The pathogen-specific results are highly consistent with those gained by using standard methods. However, standard methods should still be applied since 6 to 25% of the pathogens causing bacterial meningitis are different from those detected in the test systems. The pathogens to be expected depend on the age of the patients and predisposing factors. Table 6. shows the frequencies of the pathogens underlying acute bacterial meningitis in Europe as a function of age [8, 14, 23, 43, 55, 59, 61, 76, 105, 132].

**Table 6:** Overview of bacterial pathogens as a function of age

Newborn	
*Streptococcus agalactiae* (B-Streptococcus)	50-60%
*Escherichia coli*	14-26%
*Listeria monocytogenes*	0-3.5%
*Streptococcus pneumoniae*	0-9%
Other pathogens	10-25%
Children	
*Neisseria meningitidis*	38-56%
*Streptococcus pneumoniae*	34-46%
*Haemophilus influenzae* b	2-12%
Other pathogens	6-13%
Adults	
*Streptococcus pneumoniae*	37-59%
*Neisseria meningitidis*	24-43%
*Listeria monocytogenes*	0.8-10%
*Haemophilus influenzae* b	0.02-3.7%
Other pathogens	10-17%

### Neuroborreliosis

CSF analysis is essential to determine whether the nervous system is involved in infection with Borrelia species (AWMF S3 guideline Neuroborreliose 030/071, [144]). The diagnosis may be certain, probable, or possible according to the diagnostic criteria. Re-infection with Borrelia is possible, especially in exposed persons such as forest workers or hunters. Neuroborreliosis is thought of as an acute disease which is curable, not as a chronic disease.

In general, CSF cell count varies from 50 to 500 cells/μL, mostly lymphocytes and plasma cells. The high percentage of plasma cells sometimes makes it difficult to distinguish between meningiosis lymphomatosa and neuroborreliosis [195]. Intrathecally produced IgM in early or IgG in late disease are generally found, as are oligoclonal bands on IEF [38]. PCR and other methods of detecting antigens are of little value in diagnosing neuroborreliosis as their sensitivity in CSF and other bodily fluids is low [3]. Therefore, antibody detection by ELISA and Western blot represent key techniques here [126]. Intrathecal production of specific borrelia antibodies (borrelia AI > 1.5) confirms an acute or previously acquired neuroborreliosis. In early neuroborreliosis, the chemokine CXCL13 may be of value as high levels may be seen in the CSF of untreated patients with neuroborreliosis when antibody titers have not yet risen [149].

Since intrathecally produced borrelia antibodies may persist lifelong, re-infection may be difficult to detect. The diagnosis can be made in patients with typical symptoms, elevated cell count consisting mainly of lymphocytes and plasma cells, blood-brain barrier dysfunction, and elevated CXCL13.

Lymphocyte transformation test (LTT) and PCR in serum or blood are not recommended for diagnosing neuroborreliosis (Table 7).

**Table 7:** CSF findings in patients with neuroborreliosis

Parameter	Diagnostic LP	LP after initiating antibiotic treatment	Remarks
Cell count	≤4/μL: 0% 5-30/μL: 1% > 30/μL: 99%	normalized	Increase may indicate reinfection
Cell type	- lymphocytes: - activated lymphocytes or Plasma cells: up to 20%	normalized	
Albumin ratio	< 8 × 10^−3^: 1% 8-32 × 10^− 3^: 99%	normalized	
Quantitative IgG, IgA, and IgM synthesis	IgM > 0%: 70% IgG > 0%: 20% IgA > 0%: 1%	May persist for years	
OCBs	positive in 70% of neuroborreliosis patients	May persist for years	Differentiatial diagnosis for multiple sclerosis is necessary
Borrelia AI ≥1.5	positive in > 80%	May persist for years	Not suitable in suspected reinfection
Borrelia PCR in CSF	Positive in 10-30%	Negative after appropriate antibiotic treatment	
Lactate	< 3.5 mmol/L: 95% > 3.5 mmol/L: 5%		
CXCL13	Sensitivity 80 - 100%	Normalized after antibiotic treatment	Not specific, elevated in CNS lymphoma and inflammation

### Neurosyphilis

The diagnosis of neurosyphilis is confirmed when an intrathecal antibody synthesis against *Treponema pallidum* (Tp) is detected. The specific antibody index (AI) against *Treponema pallidum* is calculated as the ratio between Tp antibodies in CSF and serum divided by the ratio of all IgG antibodies in CSF and serum.

$$ \mathrm{Tp}\ \mathrm{IgG}\ \mathrm{AI}=\frac{\mathrm{Tp}\ \mathrm{IgG}\ \mathrm{CSF}\times \mathrm{IgG}\ \mathrm{serum}}{\mathrm{IgG}\ \mathrm{CSF}\times \mathrm{Tp}\ \mathrm{IgG}\ \mathrm{serum}} $$

Mostly, TPPA and FTA antibody titers are used to calculate the AI, which normally should be 1 in absence of neurosyphilis. In Titer-based calculation, an AI of 3 or more is positive. If an IgG-EIA technique referring to a standard curve is used, AI values of 1.5 and above are positive and confirm the diagnosis of neurosyphilis. Highly sensitive EIA may also detect a positive IgM-AI of 1.5 and above.

A positive AI does not distinguish between acute or recent infection. A positive AI against *Tp* may persist lifelong. For assessment of acute infections, a combined evaluation of typical clinical symptoms, CSF findings as well asserological analysis including*Tp*, IgG immunoassay, IgG immunoblot, IgM immunoassay, IgM immunoblot, FTA abs-IgM, VDRL, and FTA Ak- IgM are recommended. In CSF, an elevated cell count of up to 100 cells/μL, predominantly lymphocytes, a disrupted blood-CSF barrier, and CXC13 may indicate acute infection [34]. For further details, see also the AWMF guidelines “Neurosyphilis” 030/101 [184] and Diagnostik und Therapie der Neurosyphilis (059/002) [40].

If AI results are inconclusive, immunoblotting techniques may be of certain value. CSF and serum should be blotted simultaneously. If Tp-specific bands such as Tp47, Tp17, TmpA, or Tp15,5 stain more intensely in CSF than in serum, intrathecal antibody synthesis and CNS infection may be presumed [135].

### Viral meningoencephalitis

- In up to 40-80% of the suspected cases of viral meningoencephalitis (ME), the underlying pathogens are not determined due to the broad spectrum of pathogens.
- In contrast to bacterial meningitis, patients with viral ME usually present 4-7 days after onset of the disease, so that the diagnostic LP is performed somewhat later. Reference should also be made here to the AWMF guidelines “Viral Meningoencephalitis” (030/100) [119] and “FSME” (030/035) [95].
- The cell count in the CSF usually shows a slight to moderate pleocytosis.
- In the early phase of infection, neutrophilic granulocytes can typically be detected in the cell samples, in addition to lymphocytes and monocytes.
- The most common pathogens are enteroviruses, followed by flavi and bunja viruses.
- Viral CNS infections with the herpes simplex group are of particular prognostic relevance.
- DNA amplification by PCR in the first 10-14 days with good sensitivity and specificity is mainly suitable to detect the causative pathogen.
- An important exception may be a negative HSV-PCR in the first 72 h after onset of symptoms: treatment should NOT be discontinued if herpes encephalitis is suspected clinically. In these cases, an MRI with typical changes in mesial temporal regions, analysis of CSF re-drawn 3 days later and positive HSV-AI in the CSF 10 to 14 days after onset of symptoms can be diagnostically helpful.
- Pathogen-specific antibody indices only become positive after about 10-14 days.

Depending on the constellation of findings (clinical findings, basic CSF findings, and suspected pathogens (see also Table 8)), virus PCR and/or virus AI (IgG and IgM) is selected to confirm the diagnosis (Table 9).

**Table 8:** Spectrum of viral pathogens and diagnostic methods

Pathogen	Diagnostic method (1st choice)	Material	Diagnostic method (2nd Choice)	Material	Reference
*Immunocompetent patients*					
Enterovirus (Echo, Coxsackie A/B)	RT-PCR Sensitivity 97% Specificity 100%	CSF Stool, rhinopharyngeal swab	Direct detection by electron microscopy	Stool	[18, 198]
Flavivirus (FSME)	Serology Sensitivity 99% Specificity 98%	Blood	RT-PCR (early Phase) AI after 10-14 days	CSF	[72]
Herpes simplex virus Type 1 & 2	DNA-PCR Sensitivity > 95% Specificity 100%	CSF	AI after 10-14 days	CSF	[63]
Varicella zoster virus	DNA-PCR Sensitivity 95% Specificity 100%	CSF	AI after 10-14 days	CSF	[104]
*Immunocompromised patients*					
Cytomegaly virus	DNA-PCR Sensitivity 99% Specificity 99%	CSF	AI after 10-14 days pp65-Antigen	CSF Blood/ CSF	[4, 19, 96]
Epstein-Barr virus	DNA-PCR Sensitivity 100% Specificity 100%	CSF	AI after 10-14 days	CSF	[80]
Human immune deficiency virus	Serology RT-PCR Sensitivity 99% Specificity 100%	Blood	AI after 10-14 days	CSF	[5]
John Cunningham virus	DNA-PCR Sensitivity 95% Specificity > 90%	CSF	AI after 10-14 days	CSF	[20]
*Other viral CNS infections*					
Adeno virus	DNA-PCR Sensitivity 100% Specificity 99%	CSF	Antigen detection	CSF	[28]
Hanta virus	DNA-PCR Sensitivity 95% Specificity 100%	CSF	AI after 10-14 days	CSF	[178]
Measles	Serology RT-PCR Sensitivity 100% Specificity 100%	Serum CSF	AI after 10-14 days	CSF	[2]
Mumps virus	Serology RT-PCR Sensitivity 90% Specificity 100%	Serum CSF	AI after 10-14 days pp65-Antigen	CSF Blood/ CSF	[148]
Polio virus	Serology RT-PCR Sensitivity 100% Specificity 100%	CSF	AI after 10-14 days	CSF	[69]
Rabies virus	RT-PCR Sensitivity 99% Specificity 99%	CSF Blood Saliva	Direct detection by electron microscopy	CSF Saliva Brain	[47]
Rubella virus	Serology RT-PCR Sensitivity 79% Specificity 100%	Serum CSF	AI after 10-14 days	CSF	[131]
Zika virus	Serology RT-PCR Sensitivity 91% Specificity 97%	Serum CSF / Urine	AI after 10-14 days	CSF	[168]

**Table 9:** Typical CSF findings in acute and subacute phases of viral CNS infections

Parameter	Findings (diagnostic LP (days 1-7), before therapy)	Findings (follow-up LP (days > 10-14), during therapy)
CSF appearance	clear	clear
Cell count (Leukocytes/μL)	5 – 1000	<< 1000
Differential cell count	lymphomonocytic, in initial phase (days 1-3) small fraction of neutrophils	lymphomonocytic
Albumin ratio (L/S **×** 10^**−3**^)	< 20	< 10
Intrathecal Ig-Synthesis	No	Yes
Total protein (mg/L)	< 1000	<< 1000
Lactate (mmol/L)	< 3.5	< 3.5
PCR in CSF	positive	negative
Antibody index	Not detectable or < 1.5	> 1.4

### Progressive multifocal leukencephalopathy

CSF investigations are essential for diagnosing progressive multifocal leukencephalopathy (PML). The detection of JC polyomavirus (JCPyV) via PCR represents diagnostic proof of PML. However, it should be kept in mind that PCR is positive in only two thirds of cases at first LP. Thus, once PML is clinically suspected, LP needs to be repeated [110]. In some 20% of cases, the PCR remains negative even after repeated investigations of the CSF. If the clinical suspicion persists, intrathecal JCPyV-specific antibody production can be measured. An elevated antibody index (AI) suggests PML [183]. The AI remains elevated for months after the first manifestation. This investigation is limited, however, because the test can only be run at a few specialized laboratories.

The CSF cell count during PML is usually normal or only slightly elevated, usually below 20 cells/μL. To interpret CSF parameters, including cell counts and blood-CSF barrier dysfunction in PML, the underlying disease predisposing development of PML needs to be considered.

After treating the predisposing immune defect (e.g., combined antiretroviral therapy [cART] in HIV/AIDS patients or stopping medication in multiple sclerosis patients being treated with natalizumab), there may be an overreacting immune reconstitution, known as IRIS (immune reconstitution inflammatory syndrome). During IRIS, the JCPyV copy number in the CSF may even rise, and the cell number and the blood-CSF barrier dysfunction, too. We refer here to the the AWMF guideline „viral meningoencephalitis "(030/100) [119] and „HIV-infection and antiretroviral therapy "(055/001) [32] (Tables 10 and 11).

**Table 10:** Overview of routine CSF parameters and approximate frequency of pathological results in PML

Parameter	1st diagnostic lumbar puncture for PML	Follow-up during therapy (IRIS) ^a^	Comment
Cell number	≤ 4/μL: 85% ≥ 5 - ≤ 50/μL: 10% > 50/μL: 5%	May increase during IRIS	Also dependent on the underlying disease and treatment.
Cell differentiation	Normal or slightly activted. Lympho-monocytic		Also dependent on the underlying disease and treatment.
Total protein/ Albumin quotient	Normal: 50% Slightly elevated: 30% Severely elevated: 20%		
Quantitative IgG-, IgA-, IgM-synthesis	IgG > 0%: 25% IgA > 0%: 0% IgM > 0%: 0%		Is highly dependent on the underlying disease^b^
OCBs	42% ^b^		Highly dependent on the underlying disease^b^
JCPyV-PCR	Positive: 70-80%	May increase during IRIS before it normalizes	At first manifestation of PML: Sensitivity: 60-90% Specificity: 100%

^a^Empirical values from own experience due to the lack of published systematic data

^b^Value from a study with primarily HIV-PML patients. In particular, the natalizumab-PML cases will show an intrathecal immunoglobulin synthesis and oligoclonal bands corresponding to the underlying multiple sclerosis

**Table 11:** Diagnostic criteria for PML (according to [20])

Certainty of PML diagnosis	Clinical features	MRI	JCPyV-PCR
Definite	+	+	+
Probable	+	–	+
	–	+	+
Possible	+	+	−/ND
	–	–	+
Not PML	–	–	–
	+	–	–
	–	+	–

*ND* Not determined

## Non-infectious inflammatory diseases

### Multiple sclerosis

- CSF diagnosis appears to be useful and is indicated for all patients with, in particular, inflammatory CNS disease or MS.
- As a rule, a single CSF examination is sufficient for patients with suspected inflammatory CNS disease or MS. In differential diagnostically difficult cases, however, a CSF examination can be helpful in the course of the disease.
- If patients with inflammatory CNS disease or MS are diagnosed with CSF, cell count, differential cell count, glucose, lactate, quotient diagrams (albumin quotient, IgG, IgA, IgM), OCBs, as well as Lues and Borrelia antibodies should be determined [7, 145].
- Although the absence of intrathecal IgG synthesis (OKBs) does not rule out MS, it should give rise to a careful review of the diagnosis [120, 159].
- The MRZ reaction is the most specific laboratory parameter for MS and can be helpful in the differential diagnosis of MS [89].
- According to the revised McDonald criteria of 2017, patients with a clinically isolated syndrome who meet the MRI criteria for spatial dissemination can be diagnosed with relapsing-remitting multiple sclerosis if isolated oligoclonal bands are detected in the CSF [169].
- In up to 50% of all cases with negative, isolated OCBs in CSF in isoelectric focusing (IEF), intrathecal IgG synthesis can be detected by kappa free light chains, MRZ reaction, or nano-OCBs by capillary IEF [150] (Table 12).

**Table 12:** Frequency of abnormal changes in routine CSF parameters in patients with multiple sclerosis [150]

Parameter	Diagnostic LP	Remarks
Cell count	≤4/μL: 40% 5-30/μL: 55% > 30/μL: 5%	Depends on LP and relapse time interval and on topography of lesion
Differential cell count	- lymphomonocytic: 100% - activiated lymphocytes or plasma cells (< 5% of all cells): 50-60%	
Albumin ratio	< 8 × 10^−3^: 90% 8-25 × 10^− 3^: 10%	Depends on LP and relapse time interval and on topography of lesion
Intrathecal IgG, IgA, and IgM synthesis in Reiber diagrams	IgG > 0%: 72% IgA > 0%: 8% IgM > 0%: 20%	In clinically confirmed MS
OCB	CSF specific OCB (Type 2 oder 3 patterns): 88-98%	
MRZ reaction	Measles: 78% Rubella: 60% Zoster: 55%	Positive in clinical definite MS, if AI > 1,4 for two of the viruses

*AI* Antibody index, *Ig* Immunglobulin, *MS* Multiple sclerosis, *OCB* oligoclonal band

### Neurolupus

CSF analysis is of little help in diagnosing neurolupus. The diagnostic criteria for systemic lupus erythematosus according to the American College of Rheumatology and the presence of one of 19 well-defined neuropsychiatric syndromes are necessary to establish the diagnosis of neuropsychiatric lupus [6]. Each CSF parameter, such as cell count, albumin ratio, or intrathecal antibody production, may be normal or abnormal. The main advantage of CSF analysis in neurolupus is that a concomitant infection of the CNS can be detected, especially in immunocompromised patients.

Data on CSF findings in neurolupus are scarce. Pathological findings such as oligoclonal bands or intrathecal antibody synthesis may return to normal during the course of the disease. This distinguishes neurolupus from MS, where CSF findings of chronic inflammation in general persist throughout the entire disease course, independen of treatment [66].

An intrathecal antibody synthesis against dsDNA antibodies is found in only 20% of neurolupus patients [146]. A polyspecific antibody synthesis, for example, intrathecal antibody synthesis against at least two antigens, represents an even rarer event (8.7%, [75]).

The presence of certain antibodies, for example, against anticardiolipin or ribosomal p-protein, as well as high interleukin-6 levels are thought to be associated with a higher risk of developing neuropsychiatric lupus, but data from the literature are divergent (Table 13).

**Table 13:** Summary of relevant CSF findings in neuropsychiatric lupus

Parameter	Diagnostic LP	Follow-up LP (under immunosuppressive treatment)	Remarks
Cell count	1-400/μL in 30 to 44% of patients		
Cell type	Lymphocytes, monocytes		
Albumin ratio	< 8 × 10^−3^: 60% 8-25 × 10^− 3^: 40%		
Quantitative IgG-, IgA-, IgM-synthesis	IgG > 0%: 30% IgA > 0%: 13% IgM > 0%: 17%		
OCB	Type 2 or 3: 30%	Change to type 1 or 4 possible	
MRZ reaction AI ≥1.5	Measles: 30% Rubella: 30% Zoster: 40%		Intrathecal dsDNA antibodies in 20%

### Polyradiculoneuritis: Guillain-Barré syndrome (GBS), Miller-fisher syndrome (MFS), and chronic inflammatory polyneuropathy (CIDP)

A typical finding in certain diseases is high protein levels in the CSF in the absence of substantial pleocytosis (dissociation cytoalbuminique). CSF protein concentrations may reach 2000 mg/l, whereas the cell count usually does not exceed 10/μL. Disruption of the blood-CSF barrier is the main reason for elevated CSF protein [26]. Albumin ratio of CSF/serum as a measure of the blood-CSF barrier function up to 200 × 10^− 3^ may be seen, predominantly in the second to fourth week of the disease. It may take weeks and months for the values to return to normal, paralleling clinical recovery. The albumin ratio may be normal in the first week of the disease and requires that a second LP be performed later in the disease course. An intrathecal antibody synthesis or oligoclonal bands only in the CSF represent unusual findings in these medical conditions. In contrast, identical oligoclonal bands in serum and CSF are seen in up to 40% of patients and indicate systemic immune system activation.

Lymphocytes and monocytes are the main cells types found in CSF. Activated B cells or even plasma cells may be found. However, granulocytes or a cell count higher than 10/μL challenge the diagnosis.

In MFS antibodies against GQ1b are frequently found exclusively in serum, but not in the CSF. The ganglioside GQ1b is mainly expressed in eye muscles. In contrast to GBS, the CSF cell count and protein may be normal in MFS [197].

In CIDP, the cell count is normal in more than 90% of cases. Rarely, the cell count can reach 10/μL. A higher cell count should call the diagnosis into question [44]. CSF protein levels in general are elevated up to 6000 mg/l in CIDP. Likewise, the albumin ratio is elevated, too. Normal albumin ratio or oligoclonal bands may be found.

### Neurosarcoidosis

Diagnosing cases of isolated neurosarcoidosis or neurosarcoidosis in patients presenting with initial neurological symptoms still represents a challenge. Isolated neurosarcoidosis may be seen in 10% of all sarcoidosis patients. Inflammatory signs of any kind in CSF are usually present. If findings from CSF analysis are completely normal, neurosarcoidosis is unlikely. However, other frequently affected organs, such as lung and skin, should be examined to search for typical signs of the disease. Appropriate techniques are CT- thorax, T4/T8 ratio from bronchoalveolar lavage, soluble interleukin-2 receptor in CSF, FDG positron emission tomography, and Gallium scintigraphy. Sometimes, a biopsy of affected brain tissue is required to establish the diagnosis. Diagnostic criteria have been established and recently confirmed [164, 196].

As only few cases of proven neurosarcoidosis have been reported, data on sensitivity and specificity of the various diagnostic procedures are scarce. The information gathered here is the result of several small case series. A systematic review of all published data from 2013 produced heterogeneous findings for most CSF parameters [186] (Table 14).

**Table 14:** CSF findings in patients with neurosarcoidosis

Parameter	Diagnostic LP	Follow-up LP (under steroid treatment)	Remarks
Cell count	0 to 575/μL < 5/μL 20% 5-30/μL 30% > 30/μL 50%	normalzed	Higher cell counts in leptomeningeal forms
Cell type	Predominantly lymphocytes		Basophilic and eosinophilic granulocytes possible
CD4/CD8 ratio in CSF	No data available		
Glucose ratio serum/CSF	< 0,4 in 50%		Glucose level may be reduced, but glucose ratio is more precise
Lactate in CSF	elevated		Few data
Albumin ratio	8-25 × 10^−3^: 25 up to 100%		Total protein is generally elevated, but albumin ratio is more precise
intrathecal IgG, IgA, and IgM-synthesis	13 to 80%		Few data, intrathecal IgA production is seen
OCB	CSF-specific OCB (type 2 or 3): 0-70%		
ACE in CSF	20%		Genotype has an impact; thus sensitivity is low
soluble IL 2 receptor in CSF	elevated		Only in active disease and untreated patients

### Autoimmune encephalitis and paraneoplastic neurological syndromes

Autoimmune encephalitis (AE) and paraneoplastic neurological syndromes (PNS) include a heterogeneous group of autoimmune diseases affecting the central and/or peripheral nervous system that are characterized by anti-neuronal antibodies in the serum and/or CSF [118].

While CSF findings have been well described for AEs (for a comprehensive overview, see Table 15), little is known about CSF findings in PNS patients. So far, the largest study demonstrated increased cell numbers in the CSF in ~ 40% of the patients, increased total CSF protein in ~ 60-70% of the patients, or isolated cases of oligoclonal bands, indicating intrathecal IgG synthesis [143]. Approximately 5-10% of PNS patients displayed a normal CSF profile [143].

**Table 15:** CSF parameter of AE

Antigen	Pleocytosis in CSF (%)	Dysfunction of the blood/CSF barrier (%)	CSF-specific OCBs (%)	Detection of disease-specific antibody		References
				Serum	CSF	
NMDAR	70-90	~ 30	50-70	(+)	+	[33, 81]
AMPAR	50-70	40-60	~ 30	+/(+)	+	[71, 93]
GABA_A_R	40-70	20-70	20-30	+	+	[141, 158]
GABA_B_R	60-70	30-40	60	+	+	[62, 70, 91, 134]
GlyR	0-40	~ 50	20-30	+	+	[29, 115]
LGI1	10-20	20-30	< 10	+	(+)	[52, 77, 82, 101, 156, 157]
CASPR2	30-70	n.b.	~ 40	+	(+)	[22, 52, 94]
DPPX	20-60	~ 30	~ 30	+	+	[13, 65, 170]
IgLON5	0-30	30-50	0-10	+	+	[53, 74, 151]
GAD65	0-20	10-30	0-70	+	+	[15, 46, 73, 112]

### Neuromyelitis optica spectrum disorders

CSF analysis plays an important role in diagnosing NMOSD and - in addition to detecting IgG antibodies against the water channel protein aquaporin-4 (AQ)4-IgG/AQP4-Ab) and magnetic resonance imaging - helps distinguish this rare disorder from multiple sclerosis (MS). This also applies to myelin-oligodendrocyte-glycoprotein (MOG) encephalomyelitis (MOG-EM), a novel entity associated with serum autoantibodies against MOG, which phenotypically overlaps with both NMOSD and MS or may present as acute demyelinating encephalomyelitis (ADEM) and encephalitis. The current diagnostic criteria, last revised in 2015, distinguish NMOSD with AQP4-IgG from NMOSD without AQP4-IgG [193] and, according to current evidence, a subgroup of AQP4-IgG seronegative NMOSD patients harbor serum antibodies against MOG (MOG-IgG/MOG-Ab) [88]. Both AQP4-IgG-seropositive NMOSD and MOG-IgG-seropositive encephalomyelitis follow a mostly relapsing-remitting disease course and - as humorally mediated autoimmune diseases - should be distinguished from MS in terms of pathogenesis, prognosis, and therapy. Rarely, antibodies targeting the astrocytic structural protein glial fibrillary astrocyte protein (GFAP) can be detected in the CSF of some patients presenting with symptoms of meningoencephalomyelitis [49, 92].

While the typical abnormalities of CSF cells and proteins generally remain relatively stable over the entire duration of the disease in MS, pathological findings in NMOSD can often only be detected during acute attacks (20-30%) [84–86]. This applies both to CSF cell count and CSF-specific oligoclonal bands (OCB) [7]. Intrathecal IgG synthesis as detectable by using quantitative methods is observed even less frequently than CSF-restricted OCB. The MRZ reaction is mostly negative. CSF cytology also helps to distinguish the two disorders as both neutrophils and eosinophils are often identified in NMOSD, yet are absent in MS. Occasionally, very high cell counts may mimic bacterial meningitis [87]. CSF lactate levels are increased in some cases, particularly in patients presenting with acute NMOSD myelitis. Notably, a normal CSF profile does not exclude a disorder prompted by AQP4-IgG or MOG-IgG. In MOG-EM CSF, cell and protein profiles appear to be similar to those found in NMOSD [90]. CSF abnormalities are currently being investigated in larger cohorts (Table 16).

**Table 16:** Overview of relevant CSF parameters and frequency of abnormalities during relapse and in remission

Parameter	Relapse	Remission	Remarks
NMOSD with AQP4-IgG^a^			
Cell count	> 4/μL: ca. 60 - 78% > 100/μL: ca. 6%	Mostly normal (> 5/μL: 20%; > 100/μL: 0%)	Negative correlation between cell count and time (in days) since onset of relapse
Cell profile	- lymphocytes and monocytes (97% of cells) - neutrophils in 40-60% (rarely dominant cell population) - eosinophils in 10-15% - basophils in 2-4% - activated lymphocytes or - plasma cells in up to 20% (up to approximately 15% of all cells)	Less pathologically altered to normalized	
Albumin quotient	Increased in 55% (mostly 8-25, rarely > 25)	30%	
Intrathecal IgG, IgA, IgM synthesis	QIgG > Qlim: 8% QIgA > Qlim: 6% QigM > Qlim: 13%	0% 0% 0%	
OCB	CSF-restricted OCB (type 2 or type 3): 20-30%	9%	No significant difference between AQP4-IgG-positive and AQP4-IgG-negative patients
MRZ reaction AI ≥1.5 for at least two of the viruses	Almost always negative	Almost always negative	
Lactate	43%	~ 0%	
MOG-EM			
Cell count	≤5/μL:30-67% > 5/μL:33-70% > 100/μL6-28%		see ^a^
Cell profile	- lymphocytes and monocytes - plus neutrophils in 64% of cases with pleocytosis		Pleocytosis more frequent in patients with myelitis as first manifestation
Albumin quotient >Qlim(Alb):	>Qlim(Alb):32%		More common in patients with myelitis or brain stem encephalitis
Intrathecal IgG, IgA, IgM synthesis	QIgG >Qlim: 7%		Investigated in one study only
OCBs	CSF-restricted OCB (type 2 or type 3): 6-22%		
MRZ reaction AI ≥1,5	Negative		Investigated in a small cohort only

^a^In NMOSD with AQP4-IgG cell count, QAlb, QIgG, total protein, and lactate are more frequently increased and the increase is more pronounced in acute myelitis than in acute optic neuritis

## Degenerative disorders

### Dementia

In addition to anamnesis, clinical and neuropsychological examination, and cerebral imaging (described in detail in the S3 guideline “Dementia” (038/013) [35], diagnostic testing of the CSF plays an important role in the differential diagnosis of dementia. In comparison to the criteria for diagnosing Alzheimer’s Disease (AD) published in 1984 [116], neurochemical dementia diagnostics (NDD) has developed from a purely negative to a positive diagnosis. On one hand, CSF diagnostic tests in dementia syndromes serve to exclude secondary causes of dementia (e.g., inflammatory or autoimmune causes, negative diagnosis); on the other hand, specific neuropathological correlates of the primary causes of dementia can be evaluated.

Primary neurodegenerative dementias include AD, the behavioral variant of frontotemporal dementia (bvFTD), primary progressive aphasia (PPA), which can be divided into the nonfluent-agrammatic, semantic, and logopenic variants [60], corticobasal degeneration, as well as Lewy body or Parkinson’s dementia and Creutzfeldt-Jakob disease or prion diseases (see Table 17).

**Table 17:** Expected patterns of the CSF biomarkers in different neurodegenerative disorders

	Ab42 or Ab42/40	Tau	pTau
Alzheimer’s disease	↓	↑	↑
Vascular Dementia	↔	(↑)	(↑)
bvFTD	↔	(↑)	(↑)
nf-avPPA	↔	(↑)	(↑)
svPPA	↔	↔	↔
lvPPA	↓	↑	↑
CBD	(↔)	(↑)	(↑)
DLB	(↔)	(↑)	(↑)

Currently, Amyloid-β1-42 (Aβ1-42), Aβ42/40, Tau and Phospho-Tau-181 (pTau), and 14-3-3 protein and the PrPSc aggregation assay are considered clinically validated and established biomarkers ([31, 64, 114, 133, 154, 109]) and can be used mainly for positive diagnosis. However, for other primary dementias, such as PPA or DLB, there is a significant overlap of some biomarkers, especially Aβ1-42 and Tau [21]; thus, a purely neurochemical differentiation of the different etiologies based on these CSF biomarkers alone is insufficient.

Several studies have shown that the concentration ratio of Aβ1-42 to Aβ1-40 (Aβ1-42/1-40) [192] shows much better correlation with prognosis of the development of dementia, compares better with amyloid β-PET, and correlates better with postmortem validation than Aβ1-42. Therefore, it is recommended to use the Aβ42/40 ratio instead of Aβ1-42 alone [11, 12, 41, 106, 108, 129, 181].

#### Relevant biomarkers

Routinely performed basic CSF parameters: cell count, CSF differential cytology, glucose and lactate concentrations, quotient diagrams (Albumin, IgG, IgA, IgM - Quotients), and OCB by isoelectric focusing.

Specific parameters when AD is suspected: Aβ1-42, Aβ1-40, Tau, and pTau181.

Specific parameters when CJD is suspected: 14-3-3 protein, Tau, and PrPSc-aggregation assay (RT QuIC). If sporadic CJD is suspected, a stepwise approach is recommended for economic reasons, performing the PrPSc aggregation assay (RT QuIC) only when the 14-3-3 protein test is positive.

To optimize a diagnosis-oriented interpretation as well as to enable inter-center comparison of the results, diagnosis-oriented interpretation algorithms are recommended, for example, the Erlangen Score (ES [107];).

The ES combines the results of the biomarkers of amyloidosis (Aß1-42 and Aß1-42/1-40) and the biomarkers of neurodegeneration (Tau and pTau) into a five-step ordinal scale. This is particularly relevant, as common reference values for AD biomarkers are currently unavailable. Each laboratory should develop and validate its own specific reference values, which should be continuously verified in quality control schemes.

### Amyotrophic lateral sclerosis

In the basic CSF analysis, cell count, blood-CSF barrier function, and humoral signs of inflammation are usually normal [165]. Therefore, detection of neurofilaments in CSF and serum provides a useful biomarker for the early diagnosis and prognostic assessment of motoneuron diseases [27, 103, 160, 174, 176]. Currently, the light chain neurofilaments (Nf-L) and the phosphorylated heavy chain neurofilaments (pNf-H) can be determined in the CSF. For differential diagnosis of motoneuron disease, a diagnostic sensitivity of 77-83% and diagnostic specificity of 75-85% could be achieved [48, 162, 187]. Highly increased values could also be observed in CJD [161].

Similarly, good diagnostic values can also be achieved if Nf-L is measured in serum [176]. The commercially available SIMOA method (digital ELISA) is currently used to measure Nf-L in serum. Determining pNf-H in the blood with a conventional ELISA is clearly inferior to measuring Nf-L.

### Normal pressure hydrocephalus

The CSF opening pressure is usually normal (< 20 cm H_2_O) in normal pressure hydrocephalus (NPH). However, characteristic fluctuations develop over the long term (see AWMF guideline “normal pressure hydrocephalus” (030/063) [138].

Studies investigating the relevance of degeneration markers in the differential diagnosis for other dementia syndromes and gait disturbances are summarized in Table 18.

**Table 18:** Differentiation of NPH versus other dementias and controls

	Sensitivity	Specificity
Aß_1-42_	0.813	0.506
Total-Tau	0.828	0.842
Phospho-Tau	0.943	0.851

Chen et al. [30] conducted a meta-analysis of 10 studies with a total of 413 NPH patients, 186 Alzheimer’s patients, and 147 healthy controls. There was a significantly lower total-Tau and phospho-Tau in patients with NPH as compared to Alzheimer’s patients and healthy controls. NPH patients have significantly lower Aß_1-42_ concentrations in the CSF than healthy controls and slightly higher Aß_1-42_ levels than Alzheimer’s patients. Nevertheless, sensitivity and specificity are not high enough for reliable differentiation.

## Vascular diseases

### Subarachnoid hemorrhage

If a subarachnoid hemorrhage (SAH) is presumed according to clinical symptoms, but has not been proven by a CT or MRI scan, LP is necessary (see guidelines AWMF registration number: 30/073 [163, 172];). In emergency care, an evenly hemorrhagic CSF in a 3-tube test and xanthochromia of the CSF supernatant constitute leading signs.

Cytological detection of erythrophagocytosis (first, erythrophages followed by siderophages) is most specific, also enabling chronological assessment (see Table 19). The high sensitivity of an increase in ferritin at a rather high specificity is suitable for excluding SAH [127].

**Table 19:** Time course of various CSF alterations after SAH

	< 12 h	12 h – 3 d	>3d
Pleocytosis	+++	+	
Erythrocytes	+++	++	+
Oxy – Hb	+	+++	+
Erythrophages	+	++	
Bilirubin	(+)	++	+++
Siderophages		+	++
Ferritin	+	++	+++
Bilirubin Crystals		(+)	++

A CSF that is not visibly hemorrhagic (erythrocytes < 1000/μL) does not exclude a SAH, particularly in cases of smaller or older hemorrhage. Both xanthochromia and erythrophagocytosis may still be lacking in acute cases (< 12 h; sometimes, ferritin may also begin to increase later.

## Neoplastic diseases

### Neoplastic meningitis

The gold standard for diagnosing neoplastic meningitis remains CSF analysis with cytomorphological examination, in some cases followed by immunophenotyping [191]. Despite the high sensitivity of MRI scans in cases of carcinoma and elevated CSF cell count, however, differentiated CSF analysis is still indispensable when cell count is normal or to confirm hematological neoplasia [83, 98, 123]. See also AWMF-guidelines „Brain metastases and neoplastic meningitis "(030/060) [185] and „Primary CNS lymphomas "(030/059) [153].

In contrast to CSF cytology, differentiating proteins does not provide a specific diagnosis; exceptionally, however, determining tumor markers may increase the sensitivity or specificity (e.g., CEA) [190]. As a rule, malignant cells in carcinomas can easily be recognized by experienced cytologists. A sensitivity of 70-80% is reached in the first LP [191], one of around 90% for acute leukemias accompanied by high cell counts. However, in other hematological neoplasias, it may be difficult to distinguish malignant cells from inflammatory alterations based on cytomorphology alone [139]. Therefore, in these cases, immunophenotyping may be helpful particularly in known neoplasias and their surface antigens, as well as detecting monoclonality in unclear lymphocytic CSF reactions. Again, atypical cells of unknown origin may be traced to a primary neoplasia by immunophenotyping.

It is of particular relevance to distinguish lymphomatous meningitis from inflammatory lymphocytic reactions [189]. The most important single analysis is the light chain ratio of B cells to detect monoclonality in the more frequent low-grade B-NHLs. Inflammatory CSF reactions as a rule contain only few B cells. A lymphomatous meningitis in low-grade, peripheral T-NHL is rare. A summary of different immunological signs of lymphoma cells in CSF is given in Table 20.

**Table 20:** Immunological recognition of lymphoma cells in CSF

B-NHL	T-NHL
Predominance of B cells	Strong deviation of CD4/CD8-ratio, High percentage of CD4^+^CD8^+^ cells
Light chain restriction (Monoclonality) Lack of light chains Isolated IgM production	Loss of normally expressed antigens (e.g., CD7, CD5)
Co-expression of immature or aberrant antigens on or in B cells (e.g. CD 34, CD 10, CD 30, TdT, CD5)	Co-expression of immature or aberrant antigens on or in T cells (e.g.CD34, CD 10, CD 30, TdT, CD1a)

## Others

### CSF fistula

To confirm a CSF fistula, prostaglandin D synthetase (beta trace protein, BTP) or beta2 transferrin are measured. Both proteins are found in abundance in CSF, whereas concentrations in most other bodily fluids and tissue are very low. Levels above a given threshold indicate the presence of CSF. Under certain conditions, false-negative or -positive results may be obtained. In case of renal failure, for instance, BTP in other bodily fluids are higher than usual, leading to false-positive results [117]. If CSF leakage is proven by laboratory methods, it may be challenging to localize the leak. Imaging techniques such as cranial CT, cranial MRI, cisternography, or endoscopy with or without fluorescin are used [25, 180]. For details, see also AWMF guideline 039/93 “Algorithmen für die Durchführung radiologischer Untersuchungen der Kopf-Hals-Region”. More details on how to deal with CSF leaks after LP (postpuncture syndrome) can be found in the AWMF guideline 030-113 “Diagnostik und Therapie des postpunktionellen und spontanen Liquorunterdruck-Syndroms”.

Detection of glucose is of little value to prove or rule out CSF leakage [113]. Other CSF proteins such as cystatin C or transthyretin are less suitable for detecting CSF fistula (Table 21).

**Table 21:** Reference ranges of proteins for detection of CSF fistula

Parameter	Reference range	Remarks
Beta-Trace Protein (mg/l)	Serum: 0.3–0.9 CSF: 8.9–29.2 Cut-off: 1.1	Cave: renal insufficiency, endolymph fistula
Beta2-Transferrin	Qualitative detection	Cave: blood contamination

### Idiopathic intracranial hypertension

For a diagnosis of idiopathic intracranial hypertension (IIH) biochemical and cytological CSF parameters must be normal. See also the AWMF guideline “idiopathic intracranial hypertension” (030/093) [194]. In contrast, signs of elevated intracranial pressure in the absence of focal neurological signs or other causes of increased intracranial pressure are obligatory [50]. LP should be performed with the patient in a lying position to measure the opening pressure [9]. Some authors propose different cut-off values for the opening pressure depending on the body weight:

- BMI < 30: >200 mmH_2_O
- BMI > 30: > 250 mmH_2_O

These values are based on the CSF opening pressure and, thus, CSF for biochemical and cell analysis should be drawn after measuring the pressure. Pressure values taken after withdrawal of CSF are not reliable. The lowering of the CSF pressure depends on the rate of withdrawal. Withdrawal of 20 ml CSF lowers the pressure between 92 mm H_2_O (5 ml/min) and 52 mm H_2_O (1 ml/min). The needle caliber used for LP may also affect the opening pressure (Table 22).

**Table 22:** CSF pressure is dependent on the caliber of the needle used for puncture

Author	Subjects (n)	Population	Needle	Median	SD	Range	2.5	97.5
				mmH_2_O				
[58]	31	Students, healthy	22 und 26 G	145 (22G); 157 (26G)	37 (22G); 36 (26G)	85 -230 (22G); 80 – 240 (26G)	40 (22G); 50 (26G)	250 (22G); 260 (26G)
[188]	354	Neurological diseases without increased intracranial pressure	20 and 22 G, atraumatic	170		90-280	100	250

If IIH is clinically suspected and no elevated pressure is measured on the initial LP, the pressure should be measured continuously to detect B and plateau waves [171, 182].

## Data Availability

Data sharing not applicable to this article as no datasets were generated or analysed during the current study.
